# Role of CD39 in COVID-19 Severity: Dysregulation of Purinergic Signaling and Thromboinflammation

**DOI:** 10.3389/fimmu.2022.847894

**Published:** 2022-01-31

**Authors:** Elena Díaz-García, Sara García-Tovar, Enrique Alfaro, Ester Zamarrón, Alberto Mangas, Raúl Galera, José Juan Ruíz-Hernández, Jordi Solé-Violán, Carlos Rodríguez-Gallego, Ana Van-Den-Rym, Rebeca Pérez-de-Diego, Kapil Nanwani-Nanwani, Eduardo López-Collazo, Francisco García-Rio, Carolina Cubillos-Zapata

**Affiliations:** ^1^ Respiratory Diseases Group, Respiratory Service, La Paz University Hospital, Instituto de Investigación Biomédica del Hospital Universitario la Paz (IdiPAZ), Madrid, Spain; ^2^ Biomedical Research Networking Center on Respiratory Diseases (CIBERES), Madrid, Spain; ^3^ Department of Internal Medicine, Gran Canaria Dr Negrín University Hospital, Gran Canaria, Spain; ^4^ Intensitive Care Medicine, Gran Canaria Dr Negrín University Hospital, Gran Canaria, Spain; ^5^ Departament of Immunology, Gran Canaria Dr Negrín University Hospital, Gran Canaria, Spain; ^6^ Department of Clinical Sciences, University Fernando Pessoa Canarias, Las Palmas de Gran Canaria, Spain; ^7^ Laboratory of Immunogenetics of Human Diseases, La Paz University Hospital, Instituto de Investigación Biomédica del Hospital Universitario la Paz (IdiPAZ), Madrid, Spain; ^8^ Interdepartmental Group of Immunodeficiencies, Madrid, Spain; ^9^ Department of Intensive Medicine, La Paz University Hospital, Madrid, Spain; ^10^ The Innate Immune Response Group, La Paz University Hospital, Instituto de Investigación Biomédica del Hospital Universitario la Paz (IdiPAZ), Madrid, Spain; ^11^ Faculty of Medicine, Autonomous University of Madrid, Madrid, Spain

**Keywords:** COVID-19, thromboinflammation, CD39, purinergic dysregulation, hypoxia

## Abstract

CD39/NTPDase1 has emerged as an important molecule that contributes to maintain inflammatory and coagulatory homeostasis. Various studies have hypothesized the possible role of CD39 in COVID-19 pathophysiology since no confirmatory data shed light in this regard. Therefore, we aimed to quantify CD39 expression on COVID-19 patients exploring its association with severity clinical parameters and ICU admission, while unraveling the role of purinergic signaling on thromboinflammation in COVID-19 patients. We selected a prospective cohort of patients hospitalized due to severe COVID-19 pneumonia (n=75), a historical cohort of Influenza A pneumonia patients (n=18) and sex/age-matched healthy controls (n=30). CD39 was overexpressed in COVID-19 patients’ plasma and immune cell subsets and related to hypoxemia. Plasma soluble form of CD39 (sCD39) was related to length of hospital stay and independently associated with intensive care unit admission (adjusted odds ratio 1.04, 95%CI 1.0-1.08, p=0.038), with a net reclassification index of 0.229 (0.118-0.287; p=0.036). COVID-19 patients showed extracellular accumulation of adenosine nucleotides (ATP and ADP), resulting in systemic inflammation and pro-coagulant state, as a consequence of purinergic pathway dysregulation. Interestingly, we found that COVID-19 plasma caused platelet activation, which was successfully blocked by the P2Y_12_ receptor inhibitor, ticagrelor. Therefore, sCD39 is suggested as a promising biomarker for COVID-19 severity. As a conclusion, our study indicates that CD39 overexpression in COVID-19 patients could be indicating purinergic signaling dysregulation, which might be at the basis of COVID-19 thromboinflammation disorder.

## Introduction

Ectonucleoside triphosphate diphosphohydrolase 1 (NTPDase-1 or CD39) is expressed in the surface of several cells, including platelets, leukocytes and endothelial cells (1). In addition, a soluble catalytically active form of CD39 circulates in human blood (2). CD39 constitutes a major regulator of purinergic signaling, a form of extracellular signaling mediated by purine nucleotides and nucleosides such as adenosine triphosphate (ATP), adenosine diphosphate (ADP) and adenosine (ADO) (3) that exert multiple functions in inflammation and coagulation as autocrine and paracrine signaling molecules (4). For instance, extracellular ATP (eATP) is recognized by the purinergic receptor P2X_7_ (P2X_7_R) which triggers the activation of NLRP3 inflammasome which not only results in inflammation but also in increased release of tissue factor (TF) (5), the latter involved in coagulation cascade initiation (6). Furthermore, extracellular ADP (eADP) is a major activator of platelets through P2Y_12_ receptor (P2Y_12_R) (7). Under homeostatic condition, proinflammatory and pro-coagulatory eATP and eADP are hydrolyzed to anti-inflammatory adenosine (ADO) thanks to the consecutive action of CD39 and 5’nucleotidase (CD73). Thus, CD39/CD73 axis constitutes a mechanism to ensure tissue protection under acute stress, by contributing to resolution of inflammation (8). Furthermore, CD39 is well described as a principal modulatory player in inflammation and coagulation (9, 10) and its soluble form has been previously proposed as a prognosis biomarker in chronic obstructive pulmonary disease (11). Interestingly, a recent review by Franciosi et al. (12), highlights the need for investigation about the prognostic value of CD39 levels in COVID-19 patients.

COVID-19 is caused by the severe acute respiratory syndrome coronavirus 2 (SARS‐CoV-2) responsible for the current pandemic, that has caused more than five million of deaths worldwide (13). COVID-19 patients manifest a broad spectrum of symptoms ranging from mild or no symptoms to severe pneumonia that might require ICU admission and can evolve to respiratory failure, multiorgan dysfunction and death (14). Some studies point to thromboinflammation (the coordinated activation of the inflammatory and thrombotic responses) as a major cause of disease severity in patients with COVID-19 (15). In fact, the inflammatory response in patients with severe COVID-19 is particularly striking: elevated levels of inflammatory markers (such as C-reactive protein, ferritin and various cytokines), which are associated with poor outcomes (16, 17). An intriguing finding is the correlation between elevated circulating levels of inflammatory cytokines and abnormal coagulation parameters (18). Indeed, levels of prothrombotic acute phase reactants, such as fibrinogen, and D‐dimer are increased in patients with COVID-19 (19, 20), suggesting that COVID-19 can be associated with a massive inflammatory response combined with a hypercoagulable state. Although the pathophysiology of these disturbances has not yet been defined; the role of CD39 in COVID-19 severity has been suggested in this context (9). Hence, we considered an interesting priority to unravel the role of CD39 in the pathophysiology of COVID-19, since CD39 might represent a useful predictive marker for COVID-19 severity and target for therapeutic interventions. Therefore, the aim of this study was to investigate the expression of CD39 and its involvement in clinical prognosis, as well as the effects of purinergic signaling on thromboinflammation in severe COVID-19 patients.

## Material And Methods

### Study Subjects

We recruited 75 consecutive hospitalized COVID-19 patients due to severe pneumonia. Detailed information about selection criteria is provided in the supplementary methods section of online data supplement. Patients were treated according to institutional recommendations. Blood samples for study measurements and hospital-based blood testing were obtained on the day of admission and seven days afterwards. The ratio of arterial oxygen partial pressure (PaO_2_) to fractional inspired oxygen (FiO_2_) was determined at day one and seven. Exploratory endpoints were 60-day mortality, ICU admission with intubation, mechanical ventilation, and duration of hospitalization.

As control group, 30 age- and sex-matched healthy subjects were selected, without clinical evidence of respiratory or infectious disease. Additionally, samples from 18 patients with severe respiratory failure due to influenza A pneumonia from the Doctor Negrín University Hospital (Las Palmas de Gran Canaria, Spain) were also analyzed.

### Ethics

The study was approved by local Ethics Committee (PI-4087), and informed consent was obtained from all participants.

### Protein and mRNA Quantification

Proteins were quantified by specific enzyme-linked immunosorbent assay (ELISA) kits according to manufacturer’s instructions (**Supplementary Table 4**). Inflammatory cytokines were measured by cytometric bead array (CBA) as specified supplementary methods section of in supplemental data. RNA was extracted from peripheral blood mononuclear cells (PBMCs) samples, and quantified by RTqPCR, detailed procedure can be found in supplementary methods section of supplemental data.

### Hypoxia and Plasma Stimulation Model

Healthy volunteer’s PBMCs were cultured and stimulated with COVID-19 or HC plasma and/or hypoxic conditions for 16 hours. Detailed protocol can be found in the supplementary methods section of online data supplement.

### Platelet Isolation and Stimulation

O negative blood sample from healthy volunteer was collected in sodium citrate tubes and centrifuged to obtain platelet-rich plasma (PRP). PRP was diluted 1:5 in Walsh buffer. 50μL of diluted PRP were stimulated under different conditions, see details in the supplementary methods section of online data supplement.

### Statistical Analysis

Data are presented as mean ± standard error mean (SEM) unless otherwise stated. Comparisons between groups were performed by Student’s t-test with Welch correction or ANOVA with multiple comparisons by Bonferroni test, whereas chi-squared test was used in qualitative variables. Moreover, within-subject comparisons were analyzed using Wilcoxon test. Spearman’s correlation analysis was used to evaluate the relationship between variables. Associations between anthropometric and clinical and laboratory parameters and sCD39 levels with ICU admission were analyzed by bivariate and forward stepwise multiple logistic regression. To assess whether the addition of sCD39 level to the logistic model improved the predictive power for ICU admission, we calculated the area under curve of the receiver operating characteristics curve (AUC-ROC) for independent variables and the Youden index was used to calculate optimal cut off values. The equality of AUCs was assessed by the DeLong et al. method (21). Net reclassification index (NRI) and integrated discrimination improvement (IDI) were applied to quantify the improvement contributed by this approach (22). Analyses were performed using Prism 8·0 (Graph Pad, USA), MedCalc (www.medcalc.org) and SPSS 26·0 (IBM, USA) software and a P value <0·05 was considered significant.

## Results

### Characteristics of the Study Subjects

Healthy control (HC) subjects, influenza A (H1N1) patients and COVID-19 (COV) pneumonia patients were homogeneous in sex (67, 50 and 76% males, respectively), age (51 ± 14 *vs* 50 ± 19 *vs* 55 ± 14 years, respectively) and body mass index (28·3 ± 5·4 *vs* 28·6 ± 3·2 *vs* 29·2 ± 6·5 kg/m^2^, respectively). Detailed clinical characteristics of patients with COVID‐19 or influenza A pneumonia are shown in **Table 1**. Hypoxemia was treated with oxygen therapy, administered through nasal canula, Venturi mask or high-flow nasal oxygen, non-invasive ventilation according to its severity and patient tolerance. In some COVID‐19 patients (34·6%), respiratory failure progressed, requiring intubation and mechanical ventilation, and 5 patients (6·7%) died in the 60-day follow-up period.

**Table 1:** General characteristics of the study subjects*.

	COVID-19 Patients	Influenza A Patients	Healthy subjects	p-value
Age, yr	55 ± 14	50 ± 19	51 ± 14	0.285
Sex, Male/Female	57/18	9/9	20/10	0.120
Body mass index, Kg/m^2^	29.2 ± 6.5	28.6 ± 3.2	28.3 ± 5.4	0.312
Days since onset of symptoms	8 ± 3	NA	NA	NA
Symptoms at admission				
Cough	47 (67)	17 (94)	NA	NA
Active fever	39 (55)	10 (56)	NA	NA
Dyspnea	40 (57)	18 (100)	NA	NA
Myalgia	18 (25)	6 (33)	NA	NA
Sputum production	8 (12)	13 (72)	NA	NA
Chest tightness	4 (6)	NA	NA	NA
Headache	11 (16)	2 (11)	NA	NA
Fatigue	15 (21)	9 (50)	NA	NA
Anorexia	6 (8)	NA	NA	NA
Nausea	6 (8)	1 (6)	NA	NA
Diarrhea	15 (21)	5 (28)	NA	NA
Chest pain	8 (12)	NA	NA	NA
Anosmia	7 (10)	NA	NA	NA
Comorbidities				
Hypertension	20 (26)	9 (50)	0	NA
Coronary artery disease	4 (5)	NA	0	NA
Diabetes mellitus	15 (22)	6 (33)	0	NA
Obesity	15 (20)	5 (28)	0	NA
Chronic lung disease	13 (19)	NA	0	NA
Chronic kidney disease	2 (3)	2 (11)	0	NA
Hypothyroidism	2 (3)	1 (6)	0	NA
Smoking history				
Current, n (%)	41 (59)	3 (17)	0	NA
Former, (%)	11 (16)	0 (0)	0	NA
Never, n (%)	18 (26)	15 (83)	30	NA
Pneumonia severity scores				
CURB-65	0.73 ± 0.84	2.58 ± 1.68	NA	NA
Fine risk class	2.4 ± 1.0	3.6 ± 1.2	NA	NA
Laboratory findings				
PaO_2_, mmHg	65.4 ± 13.8	74.9 ± 29.5	NA	NA
PaO_2_/FiO_2_ ratio	249.7 ± 102.4	173.9 ± 83.5	NA	NA
PaCO_2_, mmHg	34.2 ± 6.5	47.7 ± 16.3	NA	NA
White cell count, 10^3^ cells/µl	7.19 ± 4.07	9.66 ± 5.94	NA	NA
Neutrophils, 10^3^ cells/µl	5.51 ± 3.32	6.50 ± 4.21	NA	NA
Lymphocytes, 10^3^ cells/µl	1.20 ± 1.87	0.88 ± 0.34	NA	NA
Eosinophils, 10^3^ cells/µl	0.02 ± 0.03	NA	NA	NA
Platelets, 10^3^ cells/µl	230 ± 75	205.40 ± 94.06	NA	NA
Hemoglobin, g/dl	13.9 ± 1.5	12.81 ± 1.31	NA	NA
C-reactive protein, mg/l	83.4 ± 73.6	46.28 ± 70.37	NA	NA
Aspartate aminotransferase, U/l	45.4 ± 28.8	70.73 ± 131.97	NA	NA
Alanine aminotransferase, IU/l	44.6 ± 32.3	56.73 ± 110.86	NA	NA
ϒ-Glutamyltransferase, IU/l	91.3 ± 92.7	66.53 ± 52.60	NA	NA
Bilirubin, µmol/l	0.53 ± 0.24	0.39 ± 0.22	NA	NA
Albumin, g/l	4.3 ± 0.3	2.95 ± 0.40	NA	NA
Ferritin, ng/ml	841.5 ± 879.7	213.73 ± 96.85	NA	NA
Lactate dehydrogenase, U/L	307.1 ± 104.0	513 ± 329.62	NA	NA
D-dimer, ng/ml	1105 ± 1424	NA	NA	NA
Fibrinogen, mg/dl	747.8 ± 281.9	NA	NA	NA
Evolution results				
Duration of hospital admission, days	11 ± 6	21 ± 12	NA	NA
ICU admission, n (%)	26 (34.6)	18 (100)	NA	NA
Exitus, n (%)	5 (6.7)	3 (17)	NA	NA

*Values are mean ± SD or number (percentage). Comparisons between groups by ANOVA or chi-squared test. NA, not applicable; PaO_2_, oxygen arterial pressure; FiO_2_, fraction of inspired oxygen; PaCO_2_, carbon dioxide arterial pressure; ICU, intensive care unit.

### Elevated CD39 Expression in COVID-19 Patients Is Related to Hypoxemia and Antiviral Immune Response

**Figure 1:**
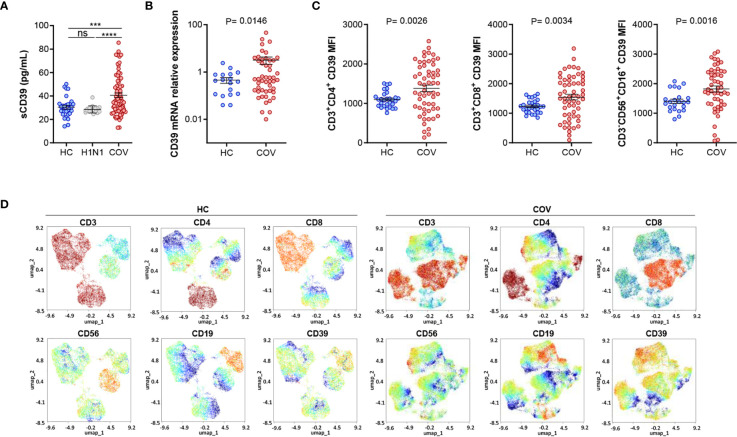
Elevated CD39 expression in COVID-19 patients. **(A)** ELISA quantification of soluble CD39 (sCD39) plasma levels in healthy controls (HC, n=30), influenza A patients (H1N1, n=18) and COVID-19 patients (COV, n=75). **(B)** CD39 mRNA expression analysis by qPCR in PBMCs from healthy controls (HC, n=22) and COVID-19 patients (COV, n=51). **(C)** Analysis of CD39 expression by flow cytometry on CD3^+^CD4^+^ and CD3^+^CD8^+^ T lymphocytes from total PBMCs of healthy controls (HC, n=29) and COVID-19 patients (COV, n=58) and CD3^-^CD56^+^CD16^+^ NK cells (HC, n=23; COV, n=51). CD39 expression is represented by MFI (Mean Fluorescence Intensity). Mean differences were analyzed using unpaired Student’s t-test analysis with Welch correction. Error bars: mean ± SEM. ***P < 0·001; ****P < 0·0001. **(D)** UMAP-guided manual gating analysis of general immune cell lineages. Healthy control PBMCs sample (HC, left panel) and COVID-19 PBMCs sample (COV, right panel). ns, non significant.

Patients with COVID-19 (COV) showed higher levels of soluble CD39 (sCD39) protein in plasma than HC or H1N1 patients (**Figure 1A**), as well as a higher CD39 mRNA expression (**Figure 1B**). CD39 surface expression was increased in CD3^+^CD4^+^ and CD3^+^CD8^+^ T cells and CD3^-^CD56^+^CD16^+^ NK cells from COVID-19 patients compared to HC (**Figure 1C**). Moreover, CD39 expression in Treg (CD4^+^CD25^+^Foxp3^+^) and monocytes (CD14^+^) cells was augmented compared to HC, while no changes in B‐cells (CD19^+^) were observed (**Supplementary Figure 1**). Additionally, Uniform Manifold Approximation and Projection (UMAP) showed CD39 expression distribution along the different immune subsets, highlighting higher expression of CD39 in COVID-19 patients in comparison with HC (**Figure 1D**).

As a consequence of hypoxemia, HIF-1α mRNA was overexpressed in COVID-19 patients with respect to HC (**Supplementary Figure 2A**). Regarding antiviral immune response, we observed elevated mRNA expression of RIG-I, MAVS, and IRF-3 in COVID-19 patients (**Supplementary Figure 2B**) evidencing intracellular recognition of the virus. Moreover, CD39 expression correlated with HIF-1 α, RIG-I, MAVS, and IRF-3 mRNA expression (**Supplementary Figure 2C**), suggesting that CD39 is associated with both hypoxia and antiviral immune response. To further support this hypothesis, we performed an *in vitro* model using isolated PBMCs from healthy volunteers stimulated with COVID-19 plasma stimulation with or without hypoxic conditions. Synergic action of COVID-19 patients’ plasma and hypoxia was needed to promote CD39 mRNA overexpression (**Supplementary Figure 2D**) as well as elevated expression of CD39 in CD4^+^, CD8^+^ T cells and CD16^+^ NK cells (**Supplementary Figure 2E**).

### Soluble Plasma CD39 Is Associated With Clinical Prognosis

**Figure 2:**
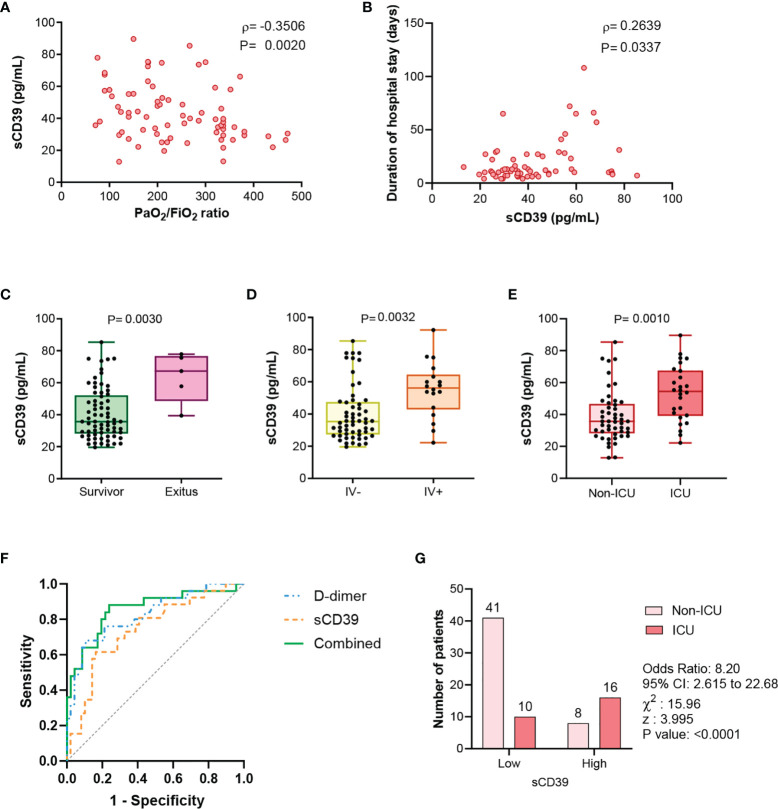
sCD39 levels are related to COVID-19 severity clinical parameters. **(A)** Correlation between PaO_2_/FiO_2_ ratio and sCD39 plasma concentration in COVID-19 patients (n=75). **(B)** At admission sCD39 plasma levels relation with duration of hospital stay (n=65). Spearman’s correlation coefficients (ρ) and P-values (P) are shown. **(C)** sCD39 plasma concentration in COVID-19 survivors (n=70) and *exitus* (n=5). **(D)** sCD39 plasma levels in COVID-19 patients non requiring invasive ventilation (IV-, n=57) and patients requiring invasive ventilation (IV+, n=18). **(E)** Comparison of sCD39 plasma levels in COVID-19 patients not derived to ICU (non-ICU, n=49) and derived to ICU (ICU, n=26). Mean differences were analyzed using unpaired Student’s t-test analysis with Welch correction. **(F)** Receiver-operating-characteristic (ROC) curve for predictive performance value for ICU admission of sCD39 (n=71), D-dimer (n=71) and the combination of both risk factors (n=71). ROC curve was assessed by Wilson/Brown test. **(G)** Contingency table comparing ICU admission for patients with sCD39 plasma levels above or below the selected cut-off point (49·52 pg/mL) (n=75). Contingency table was analyzed by Chi-squared test and cut-off value was calculated by Youden’s index.

In COVID-19 patients, sCD39 was related to hypoxemia severity, assessed by the negative correlation of CD39 with PaO_2_/FiO_2_ (ρ=-0·351, *P*=0·002) (**Figure 2A**). Moreover, plasma level of sCD39 at admission day was positively associated with the duration of hospital stay (ρ=0·264, *P*=0·0337) (**Figure 2B**). Moreover, sCD39 levels at admission were higher in patients who died (**Figure 2C**), required invasive ventilation (**Figure 2D**) or required ICU admission (**Figure 2E**). CD39 plasma levels at admission efficiently discriminated the COVID-19 patients who required ICU admission during their hospitalization, with an AUC-ROC curve of 0·732 (95%CI 0·613 to 0·883; *P*=0·0006) (**Figure 2F**) and an optimal Youden cut-off value of 49·52 pg/mL (Sensitivity 61·54%; Specificity 83·67%). COVID-19 patients with a CD39 level above this cut-off point had eight times higher risk of ICU admission (odds ratio=8·20, 95%CI 2·62 to 22·68, p<0·0001) (**Figure 2G**). In the stepwise multivariate logistic regression analysis only D-dimer and sCD39 plasmatic levels were retained as independent risk factors for ICU admission (**Supplementary Table 1**). The incorporation of sCD39 to the logistic model to estimate the risk of ICU admission determined an IDI of 0·072 ± 0·031 and a NRI of 0·229 (0·118-0·287; p=0·036), indicating that the addition of sCD39 led to a net improvement in the classification of 22·9% of the cases.

### sCD39 Reflects COVID-19 Patients’ Procoagulant and Proinflammatory State

**Figure 3:**
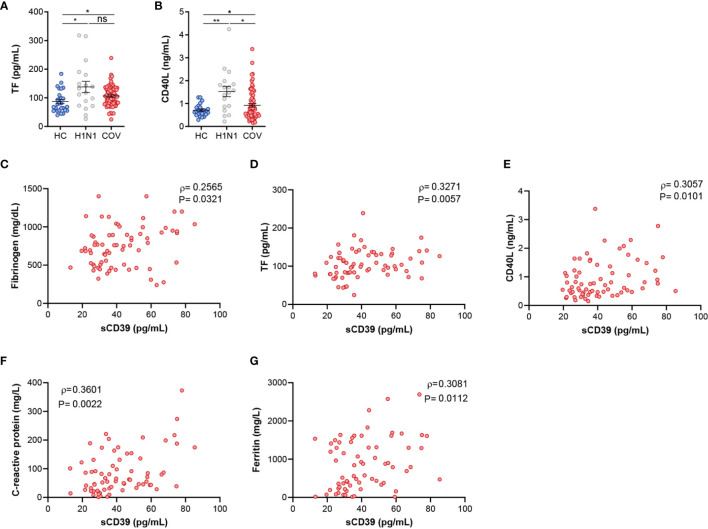
Elevated coagulation and inflammation markers in COVID-19 patients are associated with sCD39. **(A)** ELISA quantification of TF plasma levels in healthy controls (HV, n=27), influenza A patients (H1N1, n=18) and COVID-19 patients (COV, n=70). **(B)** ELISA quantification of CD40L plasma protein expression in healthy controls (HC, n=24), influenza A patients (H1N1, n=18) and COVID‐19 patients (COV, n=70). Mean differences were analyzed using unpaired Student’s t-test analysis with Welch correction. Error bars: mean ± SEM. *P < 0·05; **P < 0·01. **(C–E)** Correlations between sCD39 and **(C)** fibrinogen (n=70), **(D)** TF (n=70) and **(E)** CD40L (n=70). **(F)** Correlation between sCD39 plasma levels and C-reactive protein plasma concentration (n=70) or **(G)** ferritin plasma concentration (n=67). Spearman’s correlation coefficients (ρ) and P-values (P) are shown. ns, non significant.

To further asses CD39 role in COVID-19 severity we explored its relation with proinflammatory and procoagulant mediators. COVID-19 patients presented altered levels of fibrinogen (**Table 1**) compared to normal clinical values. We also measured plasma concentration of coagulation markers TF and CD40L. Both markers were elevated in COVID-19 patients as well as influenza A patients compared to HC (**Figures 3A, B**). Strikingly, coagulation markers were positively associated to sCD39 in COVID-19 patients (**Figures 3C** **–E**), but this association was lacking in influenza A cohort (**Supplementary Table 2**). Acute systemic inflammation markers, C-reactive protein (CRP) and ferritin were elevated in COVID-19 patients according to normal clinical parameters (**Table 1**). These markers of inflammation were also related to sCD39 plasma concentration (**Figures 3F, G**) in COVID-19 patients, but not in influenza A patients (**Supplementary Table 2**). After seven days of hospitalization, inflammation markers were ameliorated, however sCD39 plasma concentration and CD39 surface expression on immune cells as well as procoagulant markers remained elevated (**Supplementary Table 3**). To further understand CD39 role in COVID-19 severity, we studied the possible implications of purinergic nucleotides in proinflammatory and procoagulant state in COVID-19 patients.

### COVID-19-Associated Purinergic Dysregulation Is Related to TF Release and Platelet Activation

**Figure 4:**
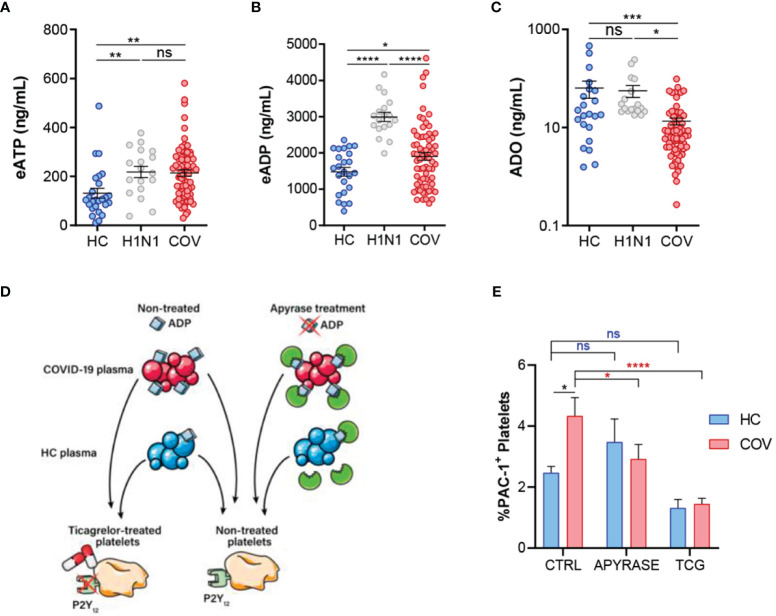
Purinergic nucleotides dysregulation and its possible role in platelet activation. **(A)** ELISA quantification of eATP plasma levels in healthy controls (HC, n=26), influenza A patients (H1N1, n=18) and COVID-19 patients (COV, n=70). **(B)** ELISA quantification of eADP in the study groups (HC, n=24; H1N1, n=18; COV, n=69. **(C)** ELISA quantification of ADO plasma levels in the study groups (HC, n=22; H1N1, n=18; COV, n=70). Mean differences were analyzed using unpaired Student t-test analysis with Welch correction **(D)** Graphic representation of *in vitro* model of platelet activation. **(E)** Flow cytometry determination of platelet activation by PAC-1 signal. Platelets were treated with healthy control plasma (blue, n=8) or COVID-19 patients’ plasma (red, n=15) under three different conditions: control (CTRL: non-treated plasmas and non-treated platelets); apyrase (APYRASE: pretreated plasmas with apyrase 0·2U/mL for 20 minutes); ticagrelor (TCG: pretreated platelets with ticagrelor 20μM for 20 minutes). Mean differences were analyzed through two-way ANOVA analysis and Sidak’s multiple comparison test. Error bars: mean ± SEM. *P < 0·05; **P < 0·01; ***P < 0·001; ****P < 0·0001. ns, non significant.

We identified higher concentrations of plasma eATP (**Figure 4A**) and eADP (**Figure 4B**) in COVID-19 and Influenza A patients compared to HC. In contrast, ADO plasma levels were reduced in COVID‐19 patients compared to influenza A patients and HC (**Figure 4C**). This could be related with reduced CD73 mRNA expression (**Supplementary Figure 3A**); as previously reported in patients with SARS-CoV-2 infection (23, 24). Altogether, these results suggest a dysregulation of the purinergic pathway leading to accumulation of eATP and eADP with reduced production of anti‐inflammatory ADO. Consequently, we decided to assess the impact of purinergic alterations in inflammation and coagulation pathways in COVID-19 patients. On one side our COVID-19 cohort presented elevated expression of NLRP3 in monocytes (**Supplementary Figure 3B**) which was associated to eATP concentration (**Supplementary Figure 3C**). As a novel finding, NLRP3 was related to TF concentration in COVID-19 patients (**Supplementary Figure 3D**), suggesting a plausible link between eATP dysregulation and TF release. On the other side, as eADP is involved in platelet activation, we elaborated an *in vitro* model to confirm the relevance of purinergic signaling in this context (**Figure 4D**), using the antibody PAC-1 which specifically recognizes an epitope on the glycoprotein IIb/IIIa complex of activated platelets. Firstly, we verified the functionality of O negative blood type healthy volunteer platelets by adding ADP at different concentrations (**Supplementary Figure 3E**). Secondly, platelets were treated with HC or COVID-19 patients’ plasma. We observed significant elevation of glycoprotein IIb/IIIa complex (PAC-1) positive platelet percentage when platelets were treated with COVID-19 plasma (**Figure 4E**). To support our hypothesis, we treated plasma with apyrase to degrade ADP and observed a reduction on platelet activation in COVID-19 plasma stimulated platelets. As a different approach, we pretreated platelets with ticagrelor (TCG), a commercial drug that blocks P2Y_12_R. Strikingly, platelets treated with ticagrelor were insensitive to COVID-19 plasma (**Figure 4E**). These data suggest that COVID-19 patients’ plasma induce platelet activation through eADP, highlighting ticagrelor as a promising therapy for COVID-19 procoagulant disorder.

## Discussion

The present study demonstrates an upregulation of CD39 in severe COVID-19 patients. Although further research is needed, our data suggest that probably CD39 expression increases in severe COVID-19 patients due to the activation of HIF-1α and RIG-I pathways. In fact, previous studies suggest that CD39/CD73 axis can be transcriptionally regulated by hypoxia through SP1 and HIF-1α (25). Furthermore, it is known that the SARS-CoV-2 nucleocapsid protein interacts with RIG-I (26) which is involved in antiviral immune response (27). Also, antiviral immune response has been associated with CD39 expression (28). Therefore, we suggest that synergic action of hypoxia and antiviral immune response in COVID-19 patients is a possible cause of CD39 upregulation.

Moreover, we identified sCD39 as a potential biomarker of COVID-19 severity. In contrast, CD39 has been reported to exert a protective role in ischemia reperfusion injury and transplantation, preventing coagulation (29). Indeed, the use of recombinant soluble CD39 has even been proposed as a therapeutic strategy for noninfectious acute lung injury among other diseases (30). Inquiringly, CD39 expression has been also reported to be increased in other respiratory diseases, such as COPD, where it has been proposed as a compensatory mechanism in response to cigarette smoke-induced lung damage (11). However, the prognostic relevance of CD39 in COVID-19 patients might be justified by its potential implication in the processes related to thromboinflammation, which are main determinants of the evolution of COVID-19 (15). In this line, the relevance of CD39 is encouraged by its relationship with several inflammatory markers such as ferritin and CRP as well as with some coagulation markers, such as fibrinogen, CD40L, and TF. These three molecules have been described to be closely related with clot formation, and the upregulation of their soluble forms in plasma has been reported in a plethora of coagulation disorders, including COVID-19 (19, 20, 31, 32). Briefly, tissue factor initiates the generation of thrombin, which not only converts fibrinogen to fibrin, but also activates platelets. Activated platelets release sCD40L to the blood stream, which in turn stimulates endothelial cell activation and secretion of tissue factor, thereby amplifying thrombosis and coagulation (32). Interestingly, various reports have recently highlighted CD39 as a key player in the crosstalk between inflammation and thrombosis (10). In addition, CD39 upregulation in COVID-19 have been suggested. For instance, Shahbazi et al., reported high levels of CD39 in CD8 T-cells from COVID-19 patients (33). Moreover, in a recent study Na Wang et al., reported high levels of CD39 mRNA in the PBMCs of COVID-19 patients, that, interestingly, were related to disease severity (34). Meanwhile, Ahmadi et al., report no significant difference in the CD39 expression on some immune subsets between COVID-19 and healthy donors (23). Therefore, additional efforts are needed in order to elucidate CD39 role in COVID-19 pathophysiology. Here we provide a more extensive study, analyzing CD39 expression in several immune subsets as well as in its soluble form. Moreover, we provide evidence relating CD39 with inflammation and coagulation markers.

According to our findings, we proposed that the molecular mechanisms underlying CD39 role on COVID-19 severity are related to impaired purinergic signaling, characterized by high levels of eATP and eADP, in combination with low levels of ADO. Interestingly, elevated eATP has been already reported in COVID-19 patients (35). eATP constitutes an important alarm for the immune system (36) as it is recognized by P2X_7_R, the most relevant purinergic receptor involved in inflammatory processes (37), such as sepsis (38). In fact, this receptor is capable of activating NLRP3 inflammasome promoting the release of pro-inflammatory and pro-coagulatory mediators (5). Thereby, inflammasome activation may constitute one angular stone in COVID-19 severity, in according with our data and previous research (39). Besides, we propose that accumulated eADP in the plasma of COVID-19 patients can activate P2Y_12_R in platelets possibly leading to the reported procoagulant disorder. Strikingly, a reversible antagonist of P2Y_12_R, ticagrelor, reduced platelet activation caused by COVID‐19 plasma. Indeed, ticagrelor can be useful to prevent sepsis-induced coagulopathy in COVID‐19 patients (40). In particular, ticagrelor not only acts on platelets by limiting its ADP‐dependent activation but can also reduce platelet-leucocyte interaction and decrease proinflammatory cytokines production (41), reducing mortality risk in patients suffering pulmonary adverse events as bacterial infection (42). In line, a retrospective observational clinical trial enrolling 1700 COVID‐19 patients concerning P2Y_12_R inhibitors (ticagrelor, clopidogrel) has been recently completed (NCT04518735). Moreover, two phase-4 trials involving clopidogrel and/or ticagrelor to treat COVID‐19 associated thrombotic events are under patients’ recruitment. First, COVID‐PACT (NCT04409834) is a multicenter, randomized-controlled trial in critically ill COVID‐19 patients involving the use of anticoagulants (including clopidogrel). Second, ACTIV‐4 study (NCT04505774) is a randomized, adaptative platform trial to compare the effectiveness of antithrombotic strategies (including ticagrelor and clopidogrel). Altogether, these proposals highlight the rising interest in P2Y_12_R inhibitors to prevent COVID‐19 associated coagulopathies.

However, our study has several limitations, which we acknowledge. First, limited sample size and follow-up time restrained the potential identification of robust prognostic events. More follow-up time will be necessary to assess the relation with different thrombotic events. Second, the results obtained allow us to suspect an association between different pathogenic pathways but do not establish a mechanistic description, since the local conditions of the pandemic restricted the procedures with infected cells. Third, this is an observational study carried out in patients with severe COVID-19 pneumonia treated according to conventional clinical practice, so the non-randomization does not allow us to infer the efficacy of different clinical approaches. And fourth, H1N1 infected patients’ biomaterial was restricted to plasma samples, preventing the realization of experiments involving immune cells.

**Figure 5:**
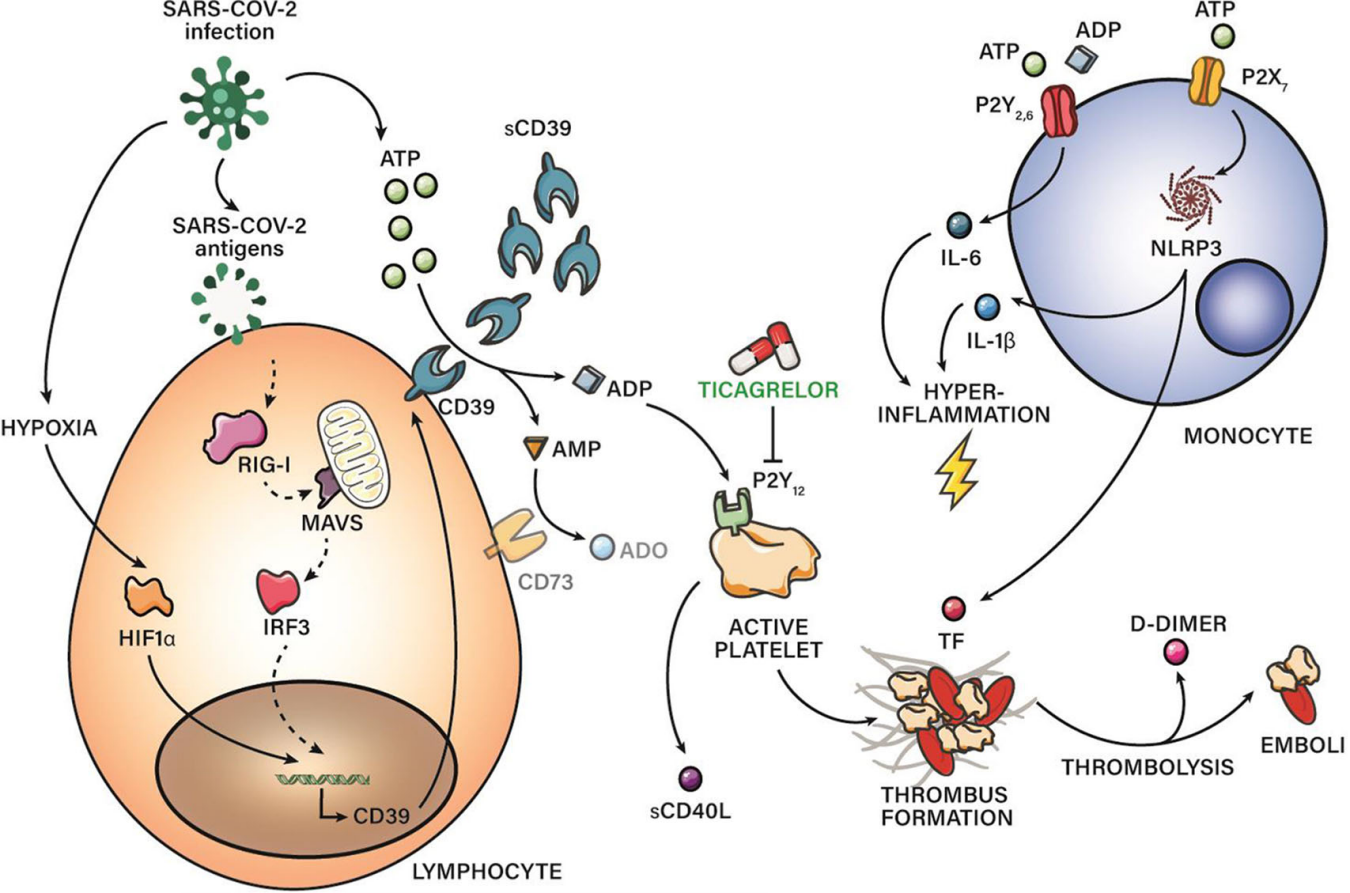
CD39 in COVID‐19 severity: schematic representation of pro-thrombotic and proinflammatory pathways related with purinergic signaling. sCD39 is upregulated along COVID-19 severity. Hypoxia and antiviral immune response through RIG-I pathway might be involved in CD39 upregulation. Moreover, this study reports impaired purinergic signaling in COVID-19, characterized by high levels of eATP and eADP in combination with low levels of anti-inflammatory ADO, maybe due to CD73 reduced expression. eATP contribute to hyperinflammation and TF release secondary to NLRP3 activation. eADP overproduction is linked to platelet activation through P2Y_12_R. Blockade of P2Y_12_R through drugs as ticagrelor is suggesting as a promising therapy for severe COVID‐19 patients.

In summary, this study reveals CD39 upregulation in severe COVID-19 patients. Moreover, the determination of its soluble form in plasma shows a certain discriminative capacity on the short-to-medium-term prognosis of these patients, so far, its usefulness as a potential biomarker could be evaluated. Moreover, this study reports impaired purinergic signaling in COVID-19, characterized by high levels of eATP and eADP in combination with low levels of ADO. The purinergic dysregulation might play an important role in modulating vascular homeostasis, inflammation, and thrombosis of patients with severe COVID-19 pneumonia. On the one hand, eATP contribute to hyperinflammation and TF release secondary to NLRP3 activation. On the other hand, eADP overproduction is linked to increased platelet activation (**Figure 5**). This observation might be exclusive for COVID-19 patients, because patients with other viral infection as influenza A, although presenting elevated proinflammatory nucleotides, maintain normal production of anti‐inflammatory ADO, and do not show elevated levels of CD39. Additional work is needed to corroborate sCD39 usefulness as a potential biomarker and to elucidate whether CD39 could be implicated in the pathogenesis of COVID-19 or whether it plays a compensatory role.

## Data Availability Statement

The original contributions presented in the study are included in the article/**Supplementary Material**. Further inquiries can be directed to the corresponding authors.

## Ethics Statement

The studies involving human participants were reviewed and approved by PI-4087. The patients/participants provided their written informed consent to participate in this study.

## Author Contributions

FG-R and CC-Z conceptualized the study. ED-G, EZ, AM, FG-R, and CC-Z advised on the study design and endpoints. ED-G, SG-T, EA, AV-D-R, RP-d-D, KN-N, EL-C, FG-R, and CC-Z performed designed experiments. EZ, AM, and RG. recruited COVID-19 patients and collect samples. JR-H, JS-V, and CR-G recruited influenza A patients and collect samples. ED-G, FG-R, and CC-Z analyzed data and performed statistical data. FG-R and CC-Z were responsible for the study management and coordination. FG-R and CC-Z drafted the paper. All authors have read and approved the final manuscript.

## Funding

This work was supported by the following fundings: Fondo de Investigación Sanitario (FIS)‐Fondos FEDER, Spain: PI19/01612 (FG-R) and COV20/00207, CP18/00028 and PI19-01363 (CC-Z).

## Conflict of Interest

The authors declare that the research was conducted in the absence of any commercial or financial relationships that could be construed as a potential conflict of interest.

## Publisher’s Note

All claims expressed in this article are solely those of the authors and do not necessarily represent those of their affiliated organizations, or those of the publisher, the editors and the reviewers. Any product that may be evaluated in this article, or claim that may be made by its manufacturer, is not guaranteed or endorsed by the publisher.

## Abbreviations

ADO, adenosine; AUC, area under the curve; COVID-19, coronavirus disease 19; CTRL,control condition; eADP, extracellular adenosine diphosphate; eATP, extracellular adenosine triphosphate; HC, healthy control; HIF-1α, hypoxia inducible factor 1α; ICU, intensive care unit; IDI, integrated discrimination increment; IRF, interferon regulatory factor; MAVS, mitochondrial antiviral signaling; NLRP3, NOD-, LRR- and pyrin domain-containing protein 3; NRI, net reclassification index; PBMCs, peripheral blood monocytes cells; RIG-I, retinoic acid-inducible gene I; ROC, receiving operating characteristics; SARS-CoV2, severe acute respiratory syndrome coronavirus 2; sCD39, soluble CD39; TCG, ticagrelor; TF, tissue factor.
