# Supplementary material for: Role of CD39 in COVID-19 Severity: Dysregulation of Purinergic Signaling and Thromboinflammation
Source: Front Immunol. 2022 Jan 31;13:847894. doi: 10.3389/fimmu.2022.847894 (PMC8841513; doi:10.3389/fimmu.2022.847894)
Supplement: Supplementary file 1 [file DataSheet_1.docx]

Supplementary Material

# Supplementary Methods

## Selection criteria for COVID-19 patients

Inclusion criteria for the COVID-19 group were: positive RT-PCR assay result from an upper respiratory tract specimen for SARS‑CoV-2; associated abnormalities or infiltrates on chest x-ray/CT scan; active or documented within 48h fever or antipyretic use; and hypoxemia (room-air oxygen saturation <92% or requirement of Supplementary oxygen). Exclusion criteria included: age <18 years; onset of COVID-19 symptoms 14 days before hospitalization; concomitant systemic bacterial or fungal infection; history of immunodeficiency or neutropenia (absolute neutrophil count <1500/mm3); active neoplasm; history of severe pulmonary disease requiring home oxygen therapy or mechanical ventilation; history of current systemic autoimmune or auto-inflammatory disease, or previous therapy with long-term oral corticosteroids, anti-IL-1β , anti-IL-6 or anti-TNFα.

## PBMCs and plasma isolation

20 mL of blood were collected by venipuncture into heparinized tubes, layered on top of 10 mL Ficoll-Paque Plus (Amersham Biosciences) and centrifuged at 1500 rpm for 20 minutes at 24°C. Plasma was removed from the upper layer, PBMCs were removed from the interphase and washed in PBS.

## Cytokines’ analysis

Inflammatory cytokines IL-1β and IL-6 concentration was measured using BD Human Inflammatory Cytokine CBA kit (551811, Becton-Dickinson Biosciences, Belgium), acquired by BD FACS-Calibur flow cytometer (Becton-Dickinson Biosciences, Belgium) and analyzed by FCAP Array software (Becton-Dickinson Biosciences, Belgium).

## mRNA isolation and quantification by qPCR

RNA was extracted using High Pure RNA Isolation Kit (Roche Diagnostics, Switzerland). 1μg of RNA was retrotranscribed using High-Capacity cDNA Reverse Transcription kit (Applied Biosystems, USA). RNA levels were measured by RTqPCR using QuantiMix Easy kit (Biotools, Spain) and Light-Cycler system (Roche Diagnostics, Switzerland) and results normalized to 18S expression. Primer sequences are listed in Supplementary Table 5 and were synthesized by Eurofins Scientific SE (Luxembourg).

## Flow cytometry

PBMCs were treated following a standard protocol using the Transcription Factor Buffer Set (Becton-Dickinson Biosciences). Cells were labeled (30 minutes, 4ºC) with antibodies detailed in Supplementary Table 6. Cells were acquired using BD FACS-Celesta flow cytometer (Becton-Dickinson Biosciences, Belgium), and data were analyzed using FlowJo vX.0.7 software (FlowJo, USA).

## Hypoxia and plasma stimulation model

PBMCs were isolated from blood samples of healthy volunteers. After isolation, 2 x 10^6^ PBMCs/well were cultured in M6 plates in RPMI medium supplemented with 100 U/mL Penicillin and 100 μg/mL Streptomycin. Cells were stimulated with 10% plasma from either HC subjects or COVID-19 patients and cultured for 16 hours in standard conditions (37°C, 21% O_2_, 5% CO_2_) or using a specific hypoxic chamber (Oxycycler C42 from [Biospherix](https://www.biospherix.com/cell-culture-equipment/oxycycler-c42), USA) which can maintain 9% O_2_.

## Platelet isolation and stimulation

O negative blood sample from healthy volunteer was collected in sodium citrate tubes and centrifuged 150g for 10min at 24º C without brake to obtain platelet-rich plasma (PRP), which was diluted 1:5 in Walsh buffer (NaCl 137mM, KCl 2.7mM, NaH_2_PO_4_ 3.3mM, MgCl_2_ 1mM and HEPES 20mM in distilled water).50μL of diluted PRP were stimulated with purified ADP (Sigma-Aldrich, ref:01905-250MG-F) or 50μL of plasma from HC or COVID-19 patients. Plasmas were untreated or treated with apyrase (0.2U/mL) (Sigma-Aldrich, ref: A6535-200UN) for 20min for ADP hydrolysis. In addition, P2Y_12_R was blocked on the surface of platelets using the commercialized drug ticagrelor (20μM) (AstraZeneca AB, Brilique) for 40min. Then platelets were stained with PAC-1_FITC_ antibody (Becton-Dickinson, ref: 340507), which recognizes glycoprotein IIb/IIIa (gpIIb/IIIa, α_IIb_β_3_) complex, a marker of activated platelets and analyzed by BD FACS-Calibur flow cytometer (Becton-Dickinson Biosciences, Belgium).

# Supplementary Figures and Tables

## Supplementary Tables

**Supplementary Table 1.** Multivariate logistic regression model including sCD39 and D-dimer as predictors of ICU admission*

|  | B | S.E. | Odds ratio | 95%CI | p-value |
| --- | --- | --- | --- | --- | --- |
| D-dimer | 0.002 | 0.001 | 1.002 | 1.001 to 1.004 | 0.003 |
| sCD39 | 0.036 | 0.021 | 1.037 | 1.001 to 1.080 | 0.038 |
| Constant | -2.128 | 1.865 | - | - | - |

*Values are adjusted by sex, age and presence or absence of obesity, hypertension or diabetes. S.E.: standard error, C.I.: confidence interval.

**Supplementary Table 2.** Spearman’s correlation of sCD39 with coagulation markers in influenza A patients

| Spearman’s correlations | TF | CD40L | CRP | Ferritin |
| --- | --- | --- | --- | --- |
| sCD39 | ρ=0.049  P=0.848 | ρ=-0.046  P=0.349 | ρ=0.198  P=0.517 | ρ=0.500  P>0.999 |

Correlations were performed by Spearman’s correlation test. Spearman correlation coefficients (ρ) and *P*-values are shown.

**Supplementary Table 3.** Comparison of different inflammatory and pro-thrombotic markers on admission and on day 7 post-admission

|  | At day 1  mean ± SEM | At day 7  mean ± SEM | Wilcoxon matched-pairs tests P-value |
| --- | --- | --- | --- |
| sCD39 (pg/mL) | 43.38 ± 3.733 | 42.42 ± 3.070 | 0.9839 |
| CD3^+^CD4^+^CD39 MFI | 1464 ± 91.47 | 1387 ± 73.13 | 0.2465 |
| CD3^+^CD8^+^CD39 MFI | 1644 ± 108.2 | 1517 ± 90.02 | 0.1572 |
| CD3^-^CD56^+^CD16^+^CD39 MFI | 1959 ± 128.4 | 1994 ± 135.0 | 0.8848 |
| eATP (ng/mL) | 212.3 ± 19.11 | 218.9 ± 17.53 | 0.5858 |
| CD14^+^ NLRP3 MFI | 1.834 ± 0.07642 | 1.987 ± 0.1336 | 0.3483 |
| C-reactive protein (mg/L) | 78.51 ± 8.939 | 19.56 ± 4.126 | <0.0001**** |
| Ferritin (mg/L) | 894.8 ± 162.1 | 630.5 ± 84.49 | 0.0330* |
| IL-1β (pg/mL) | 146 ± 87.71 | 31.67 ± 7.990 | 0.0104* |
| IL-6 (pg/mL) | 15.84 ± 8.703 | 1.412 ± 0.5005 | 0.0095 ** |
| TF (pg/mL) | 102.9 ± 6.389 | 98.17 ± 6.138 | 0.2940 |
| CD40L (ng/mL) | 0.7186 ± 0.06912 | 0.9030 ± 0.09906 | 0.1264 |

Comparisons were made by Wilcoxon matched-pairs signed rank test; P-values are shown.

**Supplementary Table 4.** ELISA kits used in the study

| ELISA target | Manufacturer | Reference | Detection limit |
| --- | --- | --- | --- |
| CD39 | CUSABIO technology, USA | CSB-EL007690 | 23.44 pg/mL |
| TF | CUSABIO technology, USA | CSB-E07913h | 3.12 pg/mL |
| CD40L | Invitrogen, Austria | BMS293 | 0.06 ng/mL |
| ATP | Cloud Clone, USA | CEA349GE | 5.25 ng/mL |
| ADP | MyBioSource, USA | MBS263784 | 0.31 ng/mL |
| ADO | MyBioSource, USA | MBS 2605344 | 0.5 ng/mL |

**Supplementary Table 5.** qPCR Primer sequences used in the study

| CD39 | Forward primer | TCGCCTCTATGGCAAGGACTAC |
| --- | --- | --- |
|  | Reverse primer | TCCAGGATGAAAGCATGGGTCC |
| RIG-I | Forward primer | ATGTGGGCAATGTCATCAAA |
|  | Reverse primer | GAAGCACTTGCTACCTCTTGC |
| IRF-3 | Forward primer | GGCTCGTGATGGTCAAGGTT |
|  | Reverse primer | CATGCCCTCCACCAAGTCCT |
| MAVS | Forward primer | CAGGCCGAGCCTATCATCTG |
|  | Reverse primer | GGGCTTTGAGCTAGTTGGCA |
| HIF-1α | Forward primer | TTCCAGTTACGTTCCTTCGATCA |
|  | Reverse primer | TTTGAGGACTTGCGCTTTCA |
| CD73 | Forward primer | TATTGCACTGGGACATTCG |
|  | Reverse primer | CCCATCATCAGAAGTGACTATG |

**Supplementary Table 6.** Flow cytometry anti-human antibodies used in the study

| Target | Fluorochrome | Manufacturer | Reference |
| --- | --- | --- | --- |
| CD39 | BB515 | BD-Biosciences | 565469 |
| CD3 | BUV395 | BD-Biosciences | 563546 |
| CD4 | PerCP-Cy^TM^5.5 | BD-Biosciences | 566923 |
| CD8 | BV711 | BD-Biosciences | 563667 |
| CD56 | BV605 | BD-Biosciences | 562780 |
| CD16 | BUV737 | BD-Biosciences | 612786 |
| CD19 | BV786 | BD-Biosciences | 563325 |
| CD25 | APC | MACS Miltenyi Biotec | 130-092-856 |
| Foxp3 | PE | BD-Biosciences | 560046 |
| CD14 | APC | Immunostep | 14A-100T |
| NLRP3 | PE | MACS Miltenyi Biotec | 130-111-209 |
| Fixable viability stain | FVS510 | BD-Biosciences | 564406 |

## Supplementary Figures

**Supplementary Figure 1.** CD39 expression in other immune-cell subsets. Analysis of CD39 expression by flow cytometry from total PBMCs of healthy controls and COVID-19 patients on CD4^+^CD25^+^Foxp3^+^ (left panel, HC=9, COV=18), CD14^+^ (middle, panel, HC=8, COV=28) and CD19^+^ (right panel, HC=14, COV=48). CD39 expression is represented by MFI (Mean Fluorescence Intensity). Mean differences were analyzed using unpaired Student *t*-test analysis with Welch correction. Error bars: mean ± SEM and *P*-values (P) are shown.

**Supplementary Figure 2.** CD39 expression is related to hypoxia and innate immune response. **(A)** HIF-1α mRNA expression analysis by qPCR in PBMCs from healthy controls (HC, n=17) and COVID-19 patients (COV, n=53). **(B)** RIG-I (left panel, HC, n=20; COV, n=52), MAVS (middle panel, HC, n=15; COV, n=33) and IRF-3 (right panel, HC, n=20; COV, n=44) mRNA expression analysis by qPCR in PBMCs. Mean differences were analyzed using unpaired Student’s *t*-test analysis with Welch correction. **(C)** Association of HIF-1α, RIG-I, MAVS and IRF3 mRNA expression with CD39 mRNA expression and sCD39 plasma concentration in COVID-19 patients. Spearman’s correlation coefficients (ρ) and *P*-values (P) are shown. **(D-E)** PBMCs from healthy subjects were isolated and cultured for 16h, either stimulated or not with 10% COVID-19 plasma and either under normoxia or hypoxia (n=6). **(D)** CD39 mRNA relative expression analysis by qPCR. **(E)** CD39 expression analysis by flow cytometry in CD3^+^CD4^+^ T-cells (left panel), CD3^+^CD8^+^ T-cells (middle panel) and CD3^-^CD56^+^CD16^+^ NK-cells (right panel) CD39 expression is represented by MFI (Mean Fluorescence Intensity). Comparisons between groups were performed by two-way ANOVA with Bonferroni’s multiple comparison test. Error bars: mean ± SEM. *: *P*<0.05.

**Supplementary Figure 3.** Dysregulated purinergic nucleotides are involved in NLRP3 overexpression. **(A)** CD73 mRNA relative expression analysis by qPCR in PBMCs from healthy controls (HC, n=14) and COVID-19 patients (COV, n=34). **(B)** Analysis of NLRP3 intracellular expression by flow cytometry in CD14^+^ monocytes from healthy controls (HC, n=18) and COVID-19 patients (COV, n=47). NLRP3 expression is represented by MFI. Comparison between groups was performed by unpaired Student t-test with Welch correction. Error bars: mean ± SEM. **(C)** Correlation of eATP plasma concentration with NLRP3 expression in CD14+ monocytes (n=47). **(D)** Correlation of NLRP3 expression in CD14^+^ monocytes with TF plasma level (n=42). Spearman correlation coefficients (ρ) and P-values are shown. **(E)** Flow cytometry quantification of percentage of PAC-1 positive platelets upon challenge with increasing concentrations of ADP or upon addition of Thrombin Receptor Activating Protein (TRAP, 10μM) (n=3). Mean differences were assessed by one-way ANOVA analysis and Tukey’s multiple comparison test. Error bars: Mean ± SEM. *: P<0.05; ****: P<0.0001 vs ADP 0 μg/mL.
